# A 44-Nucleotide Region in the Chikungunya Virus 3′ UTR Dictates Viral Fitness in Disparate Host Cells

**DOI:** 10.3390/v16060861

**Published:** 2024-05-28

**Authors:** Stephanie E. Ander, Kathryn S. Carpentier, Wes Sanders, Cormac J. Lucas, Austin J. Jolly, Cydney N. Johnson, David W. Hawman, Mark T. Heise, Nathaniel J. Moorman, Thomas E. Morrison

**Affiliations:** 1 Department of Immunology and Microbiology, University of Colorado School of Medicine, Aurora, CO 80045, USAaustin.jolly@cuanschutz.edu (A.J.J.);; 2 Department of Microbiology & Immunology, University of North Carolina at Chapel Hill, Chapel Hill, NC 27599, USA; 3 Department of Genetics, University of North Carolina at Chapel Hill, Chapel Hill, NC 27599, USA

**Keywords:** Chikungunya virus, CHIKV, 3′ UTR, viral fitness, RNA structure, SHAPE-MaP

## Abstract

We previously reported that deletion of a 44-nucleotide element in the 3′ untranslated region (UTR) of the Chikungunya virus (CHIKV) genome enhances the virulence of CHIKV infection in mice. Here, we find that while this 44-nucleotide deletion enhances CHIKV fitness in murine embryonic fibroblasts in a manner independent of the type I interferon response, the same mutation decreases viral fitness in C6/36 mosquito cells. Further, the fitness advantage conferred by the UTR deletion in mammalian cells is maintained in vivo in a mouse model of CHIKV dissemination. Finally, SHAPE-MaP analysis of the CHIKV 3′ UTR revealed this 44-nucleotide element forms a distinctive two-stem-loop structure that is ablated in the mutant 3′ UTR without altering additional 3′ UTR RNA secondary structures.

## 1. Introduction

Transmitted by infected *Aedes* species mosquitoes, Chikungunya virus (CHIKV) causes an arthritic disease that may persist or relapse in patients after the resolution of acute infection. Historically associated with Africa and Asia, CHIKV has become endemic to the Americas as of 2013 [1,2] and autochthonous transmission in Europe was first documented in 2007 [3]. As a positive-sense, single-stranded RNA virus in the *Alphavirus* genus, the 11–12 kb CHIKV genome is organized into two open reading frames flanked by 5′ and 3′ untranslated regions (UTR). The CHIKV UTRs are known to function in innate immune evasion and viral RNA replication [4]. Notably, the CHIKV 3′ UTR is one of the longest within the *Alphavirus* genus, spanning ~500–700 nucleotides [4]. 

Within the CHIKV 3′ UTR are a series of conserved, direct repeat (DR) sequences spanning 50 to 80 nucleotides that vary in copy number across CHIKV lineages; Asian genotypes contain more DR sequences than the East/Central/South African and West African genotypes. Several studies have found that deletion of DR sequences negatively impacts CHIKV infection of mosquito cells and viral dissemination in the mosquito [5,6,7,8,9,10] but has minimal impacts on mammalian infection and pathogenesis [6,7,8,9,10].

**Figure 1:**
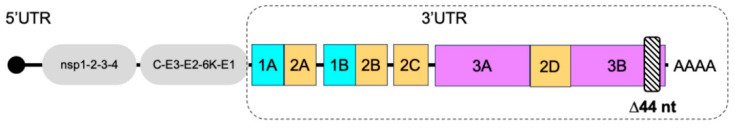
Organization of the Asian-lineage CHIKV genome with emphasis on 3′ untranslated region (UTR) elements. Diagram of the Asian-lineage CHIKV 3′ UTR highlighting the 44-nucleotide deletion, ∆44-UTR, identified in the virus isolated from the serum of persistently infected *Rag1*^−/−^ mice.

We previously reported that an Asian-lineage CHIKV isolated from the serum of a persistently infected *Rag1*^−/−^ mouse displays enhanced dissemination and pathogenicity associated with two mutations: E2 glycoprotein K200R (E2-K200R) and deletion of a 44-nucleotide region within DR 3B of the viral 3′ UTR (∆44-UTR) (Figure 1) [11]. While the E2-K200R mutation promotes enhanced viral dissemination and pathogenesis in mice through its ability to evade interactions with the scavenger receptor MARCO [12,13], we observed virulence to be further augmented by the ∆44-UTR mutation [11]. 

Here, we present a series of in vitro and in vivo viral competition assays investigating the impact of this ∆44-UTR mutation on CHIKV fitness. We find this ∆44-UTR mutation enhances viral fitness during in vitro and in vivo mammalian infections independently of the type I interferon (IFN-I) response. Meanwhile, the same ∆44-UTR mutation decreases CHIKV fitness in mosquito cells. Finally, we performed de novo RNA structural analysis on the CHIKV 3′ UTR using selective 2′-hydroxyl acylation analyzed by primer extension and mutational profiling (SHAPE-MaP). We observed that the ∆44-UTR mutation is associated with the deletion of two successive stem-loop structures but does not impact any additional RNA structures in the 3′ UTR.

## 2. Materials and Methods

### 2.1. Cell Culture and Virus Stocks

Wildtype (WT) (Balb/c) (gift from *C. Kulesza*, [14]) and type I interferon receptor knock-out (*Ifnar1*^−/−^; C57BL/6) (transformed by transfection with SV2 plasmid [15], gift from M.S. Diamond) mouse embryonic fibroblasts (MEFs) were cultured in Dulbecco’s minimum essential media (Gibco, Grand Island, NE, USA) supplemented with 10% fetal bovine serum (FBS) (HyClone, Logan, UT, USA) and penicillin/streptomycin (Gibco) at 37 °C. *Aedes albopictus* C6/36 cells (ATCC CRL-1660) were cultured in minimum essential media (Gibco) supplemented with 5% FBS, nonessential amino acids (Gibco), and penicillin/streptomycin at 28 °C. BHK-21 cells (ATCC CCL10) were cultured in *ɑ*-minimum essential media (Gibco) supplemented with 10% FBS. 

The viral mutants used in this study were generated in a cDNA clone of CHIKV strain AF15561, as described previously [11]. Based on prior studies [7], genetically marked viruses for viral competition assays were generated by site-directed mutagenesis to introduce an ApaI restriction site by silent mutation within the nsP4 gene of CHIKV (site-directed mutagenesis primers: 5′-CTAAACTAAAGGGGCCCAAAGCAGCAGCGCTGT-3′ and 5′-ACAGCGCTGCTGCTTTGGGCCCCTTTAGTTTAG-3′). Virus stocks were generated by electroporating BHK-21 cells with in vitro-transcribed RNA from cDNA clones and collection of clarified cell culture supernatants at 27 h post electroporation, as described previously [16]. Virus titers in stock aliquots were quantified by plaque assay on BHK-21 cells.

### 2.2. In Vitro Viral Fitness Competitions

To initialize in vitro viral fitness competitions, cells were inoculated with a total MOI of 1 PFU/cell (exact ratios of marked to unmarked virus are indicated as “input” in figures). Input inoculum was incubated on cells for 1 h at 37 °C, then inoculum was removed, cells rinsed twice with 1x phosphate-buffered saline (PBS), and fresh medium applied to each well. Thereafter, every 24 h for a total of 5 passages, 50 µL of cell culture supernatant from the previous day’s infections were transferred to a fresh plate of cells. The newly inoculated plate was then incubated for 1 h at 37 °C; then, inoculum was replaced with fresh medium and cells continued to be incubated at 37 °C. Following each passage of virus, remaining supernatant was collected and stored at −80 °C. 

To determine the percent virus identity (ratio of marked and unmarked virus), 50 µL of sample was spiked into 500 µL of TRIzol (Life Technologies, Carlsbad, CA, USA), and RNA was isolated according to the manufacturer’s protocol. Random hexamers were used to generate cDNA using SuperScript IV reverse transcriptase (Life Technologies) according to the manufacturer’s protocol. After generating cDNA, the nsP4 region of the viral genome was PCR-amplified using GoTaq polymerase (Promega, Madison, WI, USA) according to the manufacturer’s instructions and the following primers: 5′-ATATCTAGACATGGTGGA-3′ and 5′-TATCAAAGGAGGCTATGTC-3′. PCR products were digested with ApaI (NEB, Ipswich, MA, USA) and PspOMI (NEB) at room temperature for 30 min followed by 2–3 h at 37 °C. The redundancy of the double digestion with the neoschizomers ApaI and PspOMI ensured complete digestion of the genetically marked PCR products within each sample, as previously described [7]. Digested PCR products were analyzed on a 1% TAE gel, stained with ethidium bromide, imaged, and band intensities per lane were quantified (Syngene G Box). Marked virus generates a PCR product susceptible to restriction enzyme digestion (cleaving the PCR product in half), while unmarked virus is resistant to cleavage.

### 2.3. Mouse Experiments

Four-week-old WT C57BL/6J mice were obtained from Jackson laboratory (strain #000664) and congenic *Ifnar1*^−/−^ mice (Jackson laboratory strain #028288) were bred in a specific-pathogen-free vivarium at the University of Colorado Anschutz Medical Campus. All mouse experiments were performed in an animal biosafety level 3 laboratory. Mice were inoculated with a total of 2000 plaque-forming units (PFUs) of marked and unmarked CHIKV as indicated. Upon termination of each experiment, mice were euthanized by isoflurane sedation followed by bilateral thoracotomy; mice were perfused with PBS and collected tissues were homogenized in TRIzol (Life Technologies) using a MagNA Lyser instrument (Roche, Rotkreutz, Switzerland). RNA isolation and percent virus identity was determined as described above.

### 2.4. Determination of Viral 3′ UTR Secondary Structure

The 3′ UTR of WT and the ∆44-UTR CHIKV AF15561 were PCR-amplified from the cDNA clones using Q5 polymerase (Promega) and the following primers: 5′-AATAGAATTCTAATACGACTCACTATAGGGTTTAGCAGGCACTAACTTGA-3′ and 5′-GAAATATTAAAAACAAAATAACATCTCCTA-3′. The forward primer was designed to incorporate a T7 promoter at the start of the 3′ UTR amplicon for in vitro transcription reaction (Life Technologies, Carlsbad, USA). The resultant RNA was used for SHAPE-MaP as described previously [8].

## 3. Results

### 3.1. The CHIKV ∆44-UTR Mutation Enhances Viral Fitness in Murine Cells

**Figure 2:**
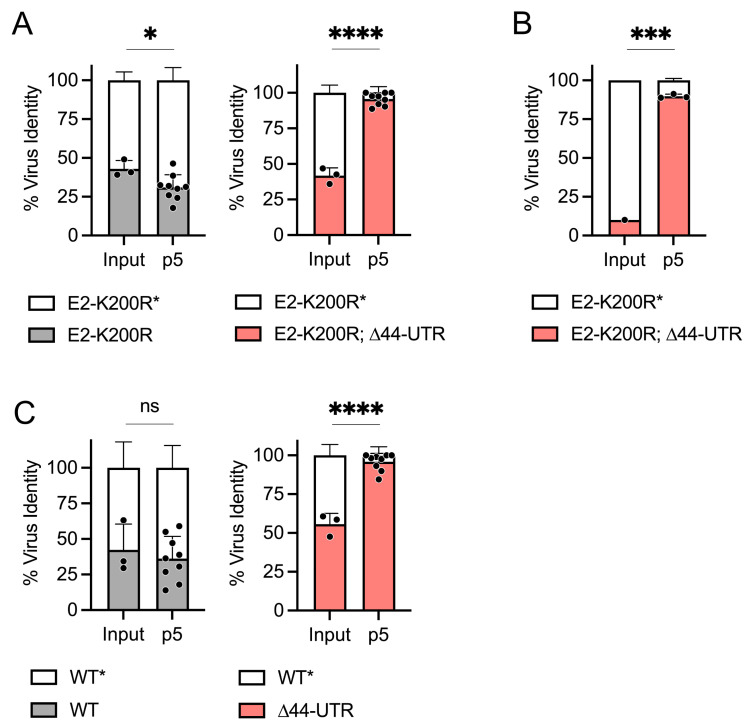
The ∆44-UTR mutation enhances CHIKV fitness in murine cells in vitro. In vitro viral fitness competitions of marked and unmarked viruses performed through five serial passages (p5) in WT MEFs: (**A**) similar starting inputs of E2-K200R* vs. E2-K200R or E2-K200R; ∆44-UTR viruses, (**B**) 10:1 starting ratio of E2-K200R* vs. E2-K200R; ∆44-UTR virus, (**C**) similar starting inputs of WT* vs WT or ∆44-UTR virus. For each sample, cDNA was synthesized from extracted RNA and used for PCR amplification of a region within the CHIKV nsP4 gene; PCR products were then digested with ApaI/PspOMI and run on an agarose gel. Virus identity is determined by the presence or absence of a synonymous genetic marking in the nsP4 gene (*) that confers susceptibility to ApaI/PspOMI restriction digest. The ratio of ApaI/PspOMI-resistant versus -susceptible PCR products was used to calculate % virus identity. Input indicates starting ratios of marked to unmarked virus in initial inoculum. Data are from 1–3 independent experiments performed in triplicate. Statistics are unpaired *t*-test; ****, *p* < 0.0001; ***, *p* < 0.001; *, *p* < 0.05; ns, not significant.

We performed a series of blind-passage viral competition assays using genetically marked (indicated by asterisk) and unmarked viruses to investigate whether the ∆44-UTR mutation found in CHIKV isolated from a persistently infected *Rag1*^−/−^ mouse [11] confers a selective fitness advantage over WT virus during infection of murine cells. We inoculated mouse embryonic fibroblasts (MEFs) with a 1:1 mixture of E2-K200R* and E2-K200R; ∆44-UTR viruses and blind-passaged infected cell supernatants every 24 h. By passage 5, the E2-K200R; ∆44-UTR mutant virus was the dominant virus in the cell culture supernatant (*p* < 0.0001); a similar shift in virus proportions was not observed in the E2-K200R* versus E2-K200R competition (Figure 2A). This selection for the E2-K200R; ∆44-UTR mutant virus was also observed when input ratios were skewed against the E2-K200R∆44-UTR mutant (Figure 2B). Evaluation of the ∆44-UTR mutation in the absence of the E2-K200R mutation revealed deletion of this 44-nucleotide region in the 3′ UTR alone strongly enhances (*p* < 0.0001) viral fitness in murine cells in vitro (Figure 2C). 

### 3.2. The CHIKV ∆44-UTR Mutation Is Deleterious in Mosquito Cells

**Figure 3:**
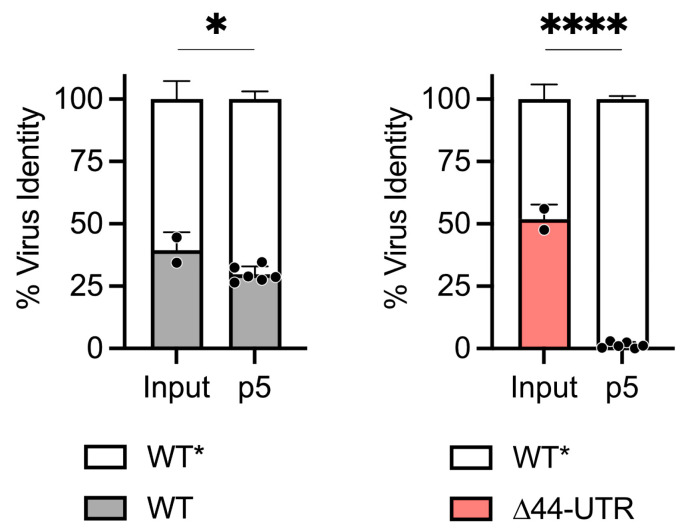
The ∆44-UTR mutation is deleterious in mosquito cells. In vitro viral fitness competitions of two viruses performed through five serial passages (p5) in C6/36 *Aedes albopictus* cells. Virus identity was determined by the presence or absence of a synonymous genetic marking in the nsP4 gene (*) that confers susceptibility to ApaI/PspOMI restriction digest. Input indicates starting ratios of marked to unmarked virus in initial inoculum. Data are from 2 independent experiments performed in triplicate. Statistics are unpaired *t*-test; ****, *p* < 0.0001; *, *p* < 0.05.

As deletions in the CHIKV 3′ UTR are usually disadvantageous in the mosquito [5,6,7,8,9], we performed viral fitness competitions in *Aedes albopictus* C6/36 cells. In direct contrast to mammalian cells, the ∆44-UTR mutant virus was significantly less fit (*p* < 0.0001) than the WT* CHIKV in C6/36 cells (Figure 3). These observations suggest opposing selective pressures operate on the CHIKV 3′ UTR during infection of mammalian versus mosquito cells.

### 3.3. The Fitness Advantage Conferred by the ∆44-UTR Deletion in CHIKV Is Independent of the Type I Interferon Response and Enhances Viral Fitness In Vivo

**Figure 4:**
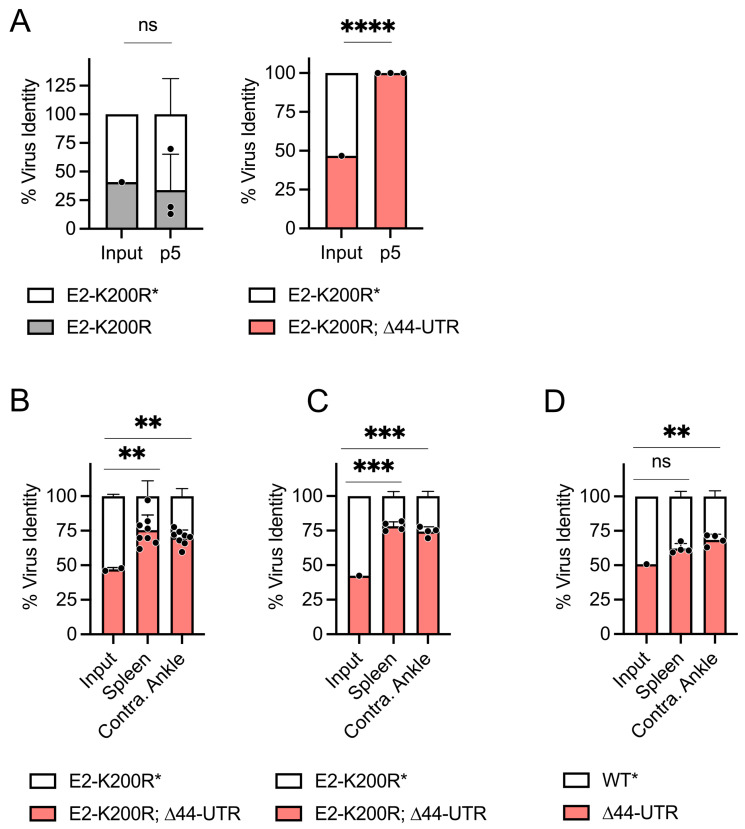
The fitness advantage conferred by 3′ UTR deletion in murine cells is independent of the type I interferon response and confers a fitness advantage in a mouse model of CHIKV disease. (**A**) In vitro viral fitness competitions of marked and unmarked CHIKV performed through five serial passages (p5) in *Ifnar1*^−/−^ MEFs. Data are from 1 experiment performed in triplicate. (**B**–**D**) For in vivo viral fitness competitions, mice were inoculated with 1000 PFU of each virus. Data are from 1–2 independent experiments performed with 4 mice per experiment. (**B**) Virus competition in WT C57BL/6 mice; virus proportions in spleen and contralateral ankle determined at 4 days post inoculation. (**C**,**D**) Virus competition in *Ifnar1*^−/−^ C57BL/6 mice; virus proportions in spleen and contralateral ankle determined at 3 days post inoculation. (**A**–**D**) Virus identity was determined by the presence or absence of a synonymous genetic marking in the nsP4 gene (*) that confers susceptibility to ApaI/PspOMI restriction digest. Input indicates starting ratios of marked to unmarked virus in initial inoculum. Statistics are unpaired *t*-test (**A**) or one-way ANOVA (**B**–**D**); ****, *p* < 0.0001; ***, *p* < 0.001; **, *p* < 0.01; ns, not significant.

We hypothesized that the RNA sequences/structures deleted in the ∆44-UTR virus could be recognized by cellular innate defenses, providing the mutant virus with a fitness advantage in the presence of an active type I interferon (IFN-I) response. To test this hypothesis, we performed viral competition experiments in *Ifnar1*^−/−^ MEFs. Even in the absence of IFN-I signaling, the E2-K200R; ∆44-UTR mutant virus significantly (*p* < 0.0001) out-competed the E2-K200R* (i.e., UTR-intact) virus (Figure 4A). These data suggest the selective pressure exerted on the CHIKV 3′ UTR during mammalian cell infection is independent of IFN-I.

We next evaluated the fitness of the ∆44-UTR virus in a mouse model of CHIKV dissemination. After inoculating WT C57BL/6 mice with an equivalent mixture of E2-K200R* and E2-K200R;∆44-UTR viruses in the left-rear footpad, we observed significantly enhanced burdens of the E2-K200R;∆44-UTR mutant virus in the spleen (*p* < 0.01) and contralateral ankle (*p* < 0.01) at 4 days post inoculation (dpi) (Figure 4B) as compared to the E2-K200R* virus. In accordance with our in vitro observations suggestive of an IFN-I-independent selective pressure, the E2-K200R;∆44-UTR mutant also exhibited enhanced dissemination (*p* < 0.001) in *Ifnar1*^−/−^ mice compared to E2-K200R* virus (Figure 4C). We also evaluated the impact of the ∆44-UTR mutation in the absence of E2-K200R, as we have previously found the single E2-K200R mutation, but not the ∆44-UTR mutation alone, significantly enhances CHIKV dissemination at 1 dpi in WT C57BL/6 mice [11,12,13]. Following co-inoculation of *Ifnar1*^−/−^ mice with equivalent doses of WT* and ∆44-UTR viruses, we found in the absence of IFN-I signaling that the ∆44-UTR constituted a significantly (*p* < 0.01) greater proportion of the viral burden at 3 dpi in the contralateral ankle as compared with WT* virus levels (Figure 4D). Thus, the ∆44-UTR mutation enhances CHIKV fitness in mammalian cells both in vitro and in vivo in an IFN-I-independent manner.

### 3.4. The 44-Nucleotide Region of the CHIKV 3′ UTR Is Characterized by Distinct Secondary Structure

**Figure 5:**
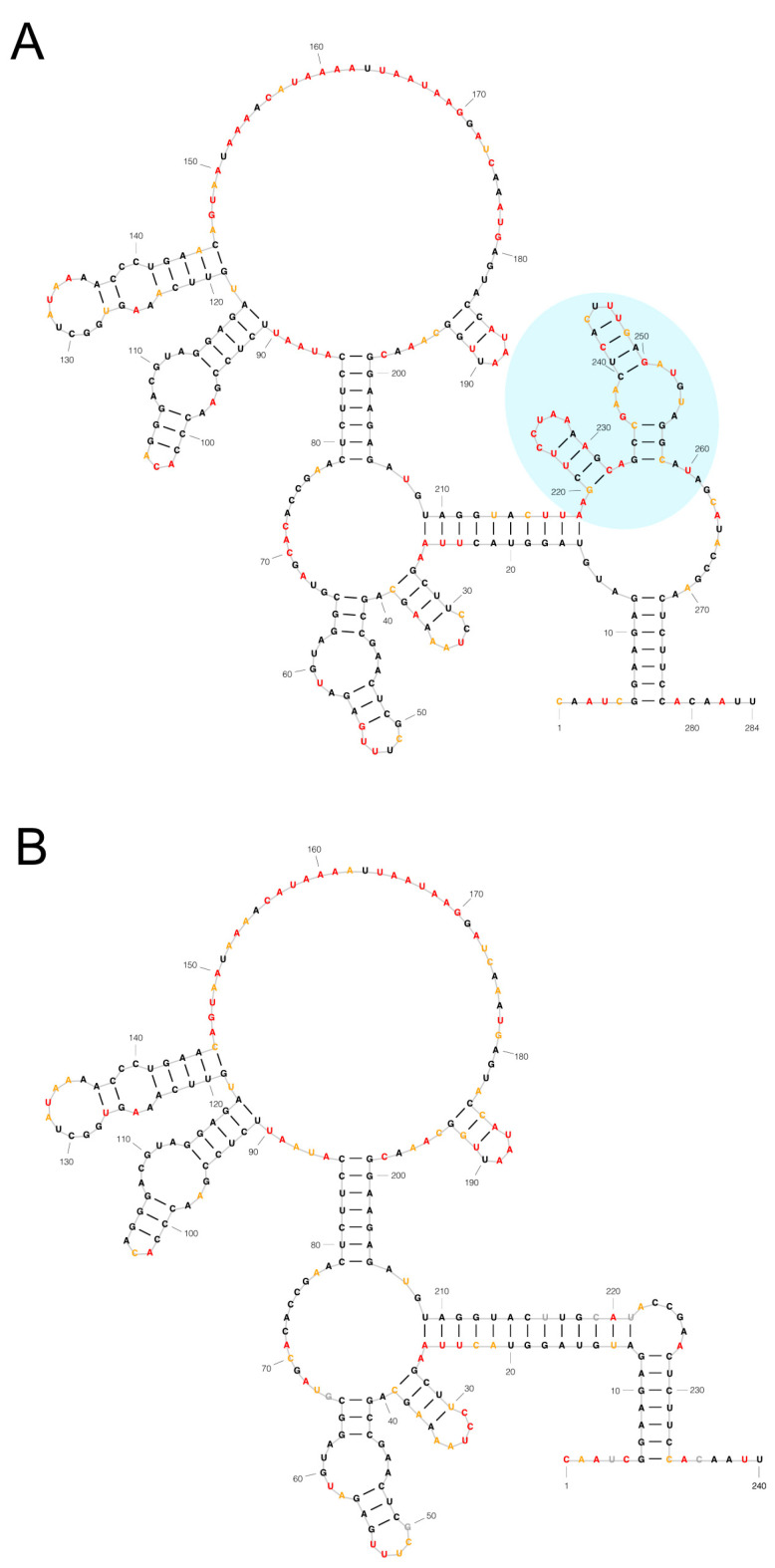
The 44-nucleotide deletion in the CHIKV 3′ UTR is characterized by distinct RNA secondary structure. SHAPE-MaP informed models of (**A**) WT and (**B**) ∆44-UTR CHIKV 3′ UTRs. The 44-nucleotide region is highlighted in blue in (**A**). The nucleotide color corresponds to the SHAPE reactivity detected. SHAPE reactivities above 0.8 indicate likely unpaired bases (shown in red). SHAPE reactivities below 0.4 indicate likely paired and therefore unreactive bases (colored black). Intermediate reactivities (e.g., 0.4–0.8) are suggestive of unpaired bases (shown in yellow).

We hypothesized this 44-nucleotide region of the CHIKV 3′ UTR may be associated with a specific RNA secondary structure. Therefore, we performed SHAPE-MaP of the WT and ∆44-UTR mutant viral 3′ UTR RNAs and used these data to construct an experimentally validated model of the RNA secondary structure. Strikingly, deletion of the 44-nucleotide region results in deletion of two successive stem-loop structures without disrupting any other RNA structures present in the CHIKV 3′ UTR (Figure 5). 

## 4. Discussion

We previously reported the naturally acquired deletion of a 44-nucleotide region in the 3′ UTR contributes to the enhanced viral pathogenesis of CHIKV in WT mice in the presence of the E2-K200R mutation [11]. This region of the CHIKV 3′ UTR exists in all CHIKV lineages; however, the Asian-lineage CHIKV is unique in possessing two copies of this region due to a genomic duplication event [7]. In this present study, we evaluated the impact of a single copy deletion of this element (specifically deletion of the element in DR3B as illustrated in Figure 1) in an Asian-lineage CHIKV (strain AF15561) on viral fitness rather than viral pathogenicity. Using viral competition assays, we found the ∆44-UTR mutation enhanced CHIKV fitness during murine infection, both in vitro and in vivo (Figure 2 and Figure 4), but decreased CHIKV fitness in *Aedes albopictus* C6/36 mosquito cells (Figure 3). As our present study focused on evaluating the specific ∆44-UTR mutation isolated from the serum of an Asian-lineage CHIKV persistently infected *Rag1*^−/−^ mouse, future studies are warranted to investigate the fitness phenotype of the ∆44-UTR mutation in DR3A of Asian-lineage CHIKV as well as in other CHIKV lineages possessing a single copy of this 3′ UTR region (East/Central/South African and West African lineages).

Our observations are supported by a recent report by Bardossy et al. [10] showing species-specific differences (mammalian versus mosquito) in viral titers following multistep viral growth curves of WT CHIKV and a mutant, Asian-lineage CHIKV strain containing deletions within the DR3A and DR3B 3′ UTR elements (inclusive of the 44-nucleotides investigated herein). Interestingly, our head-to-head competition of WT and ∆44-UTR viruses exacerbates these differences, suggesting the differential involvement of a host factor that interacts with this discrete region of the CHIKV 3′ UTR. Towards that end, we investigated whether viral evasion of the IFN-I response may contribute to the enhanced fitness of the ∆44-UTR mutant in mammalian cells. Yet, intriguingly, the ∆44-UTR mutant maintained its fitness advantage in both *Ifnar1*-deficient cells and mice (Figure 4A,C,D), suggesting WT CHIKV infection of murine cells is modulated by interactions of an IFN-I-independent antiviral host factor with the viral 3′ UTR. Furthermore, our in vivo observations that the ∆44-UTR mutation enhances CHIKV fitness lend greater significance to both our own (Figure 2 and Figure 4) and previously published in vitro data [10]. In particular, the similar, IFN-I-independent fitness advantage conferred by the ∆44-UTR mutation both in vivo and in vitro (Figure 4) supports the translatability of the in vitro models for further mechanistic investigations.

While the ∆44-UTR mutation is advantageous for CHIKV in mammalian cells, we observed this same mutation to be deleterious for viral fitness in the context of C6/36 mosquito cells (Figure 3). This observation aligns with other studies describing more stringent requirements of the viral 3′ UTR for productive CHIKV infection of mosquitoes and mosquito cells [5,6,7,8,9,10]. While our present study evaluated the fitness of the ∆44-UTR mutant, a recent report by Bardossy et al. [10] found multiple copies of the region encompassing this 44-nucleotide element enhances Asian-lineage CHIKV infection of C6/36 cells in a successively additive manner. 

Finally, our SHAPE-MaP analysis of the CHIKV 3′ UTR revealed the 44-nucleotide element under investigation forms two successive stem-loop structures (Figure 5). Moreover, we experimentally showed that deletion of this 44-nucleotide element results in precise removal of the two-stem-loop structures without disrupting any other RNA secondary structures elsewhere in the CHIKV 3′ UTR (Figure 5B). Among arboviruses, both 3′ UTR RNA sequence and structure have been shown to interact with host factors, such as the microRNA binding sites in Eastern equine encephalitis virus (mediated by viral sequence) [17] and the pseudoknots (RNA structure) in the flavivirus genome that inhibit the cellular exoribonuclease Xrn1 [18]. Future studies are warranted to determine whether the RNA sequence and/or structure of this 44-nucleotide element in the CHIKV 3′ UTR is responsible for mediating the opposing fitness phenotypes in mammalian and mosquito cells. 

## Data Availability

The data in this study can be accessed on Mendeley Data: Morrison, Tem (2024), “A 44-nucleotide region in the chikungunya virus 3′ UTR dictates viral fitness in disparate host cells”, Mendeley Data, V1, doi: 10.17632/fvgy8bh3n4.1.
